# Mental Development

**Published:** 1866-08

**Authors:** 


### MENTAL DEVELOPMENT.

THE great mind and the vigorous constitution are so often united in the same person, that we are compelled to the conclusion that high physical health in earlier life is, as a general rule, the ground-work of mental power. Some of the most eminent men of the present century are men who, in earlier life, were exposed to great physical hardships, had to endure a great deal of hard work, and to pass through many trying self-denials. Thus it is that out of the " West "—that very " West " which, in the estimation of multitudes of Eastern minds, is the abode of people, who, in morals and manners, are at no very great remove from savageism—stars have arisen, and are rising, which so beautify the mental sky above us, as to cause us to inquire, What will the end of these things be?

A correspondent of the *Philadelphia Bulletin*, writes of Rev. N. L. RICE, D. D.

"He is, unquestionably, one of the ablest of living American divines. His preaching is compact and argumentative, yet singularly lucid and simple. His manner is easy and unassuming, yet remarkably earnest and impressive. As a debater, he has few, if any, superiors in the land, as his celebrated discussions with ALEXANDER CAMPBELL, and various religious errorists, have clearly shown."

What the "Home Missionaries" had to encounter near half a century ago, is illustrated in the life of one who is now no more, as to the visible world around us:

"In the western part of Virginia was situated a log cabin, the chinks of which were daubed and filled with yellow mud. It had, perhaps, half a second story, where you could study astronomy without leaving bed, and adopt hydropathy without the aid of any doctor; the kitchen serves as a breakfast and dining, a dressing and preaching-room. A number of hens with their chickens are taken in for safe-keeping. Amid the barking of dogs and noise, and after midnight, when all had retired to rest, stretched on his stomach, before the embers of the fire, which served for his midnight oil, he not only acquired a sufficient knowledge to prosecute his calling, but became master of several languages. He preached in one year four hundred times, travelled five thousand miles; and at the end of that time his salary amounted to twelve dollars and ten cents! That man was HENRY B. BASCOM, who was since raised to the Methodist Episcopacy, in which position he was an ornament to the Church."

Not all the elegance of manner and high-bred courtesy in the world is found in the East; for the most perfect pattern of a Christian gentleman within the last fifty years, was found in the person of the Rev. JOHN C. BRECKENRIDGE, D. D., the uncle of Ex Vice-President of the United States, and the brother of the Modern JOHN KNOX, whose name, like that of HENRY CLAY, needs neither prefix nor affix to give it note, and whose fellow-citizen, and neighbor, and friend he was, ROBERT J. BRECKENRIDGE; and all these were "*Western*" men.

Never in the history of the old "Tabernacle" of Broadway, did so many hundreds of disappointed men and women go away from its doors, for want of room, for many nights in succession, as when NELSON and GALLAHER riveted the attention of the motionless thousands who hung upon their lips—men these were, as giant in body as in intellect, raised mainly on corn, potatoes, and wild meats, in the mountain fastnesses of East Tennessee.

And then there is another man, the playmate of our earliest youth, whose father wore the hunting-shirt of DANIEL BOONE, and walked in the moccasin of the wild Indian; hardy as a pine-knot is he, and one of the most efficient clergyman of his faith. The "*Presbyterian Quarterly Review*" has an article by Dr. WILSON, of Newark, which says of one of his sermons: "We cannot forbear to quote the noble peroration, *it reminds one of the swell and march of* Dr. MASON'S *Sermon on the Mediatorial Reign:* he was born and lived in the West, until his heart is in sympathy with its vastness." But the East could not as well do without this Western man; so the Rev. THORNTON A. MILLS, D. D., conducts, in New York, one of the most important offices in his church.

We write these things to show, that to be great, and to accomplish great things, to fill efficiently the most important places in the church and nation, vigorous bodily health seems almost indispensable. To all young men, then, who aim to do good on a large scale, we say most earnestly: *Nurse your constitution with pious care, invigorate it; study to be well, as the necessary means of doing well, in the highest sense of the term.*

